# Impact of Cannabinoid Compounds on Skin Cancer

**DOI:** 10.3390/cancers14071769

**Published:** 2022-03-31

**Authors:** Robert Ramer, Franziska Wendt, Felix Wittig, Mirijam Schäfer, Lars Boeckmann, Steffen Emmert, Burkhard Hinz

**Affiliations:** 1 Institute of Pharmacology and Toxicology, Rostock University Medical Centre, 18057 Rostock, Germany; robert.ramer@med.uni-rostock.de (R.R.); franziska.wendt@med.uni-rostock.de (F.W.); felix.wittig@med.uni-rostock.de (F.W.); 2 Clinic and Polyclinic for Dermatology and Venereology, Rostock University Medical Centre, 18057 Rostock, Germany; mirijam.schaefer@med.uni-rostock.de (M.S.); lars.boeckmann@med.uni-rostock.de (L.B.); steffen.emmert@med.uni-rostock.de (S.E.)

**Keywords:** cannabinoids, endocannabinoid system, skin cancer, melanoma, squamous cell carcinoma

## Abstract

**Simple Summary:**

Recent research has suggested that the endocannabinoid system offers several pharmacotherapeutic targets for drug administration as new options for the treatment and prophylaxis of skin cancer. This review focused on the anticarcinogenic mechanisms of cannabinoids at the different levels of skin cancer progression, such as inhibition of tumour growth, proliferation, invasion and angiogenesis, as well as inducing apoptosis and autophagy.

**Abstract:**

Drugs targeting the endocannabinoid system are of interest as potential systemic chemotherapeutic treatments and for palliative care in cancer. In this context, cannabinoid compounds have been successfully tested as a systemic therapeutic option in preclinical models over the past decades. Recent findings have suggested an essential function of the endocannabinoid system in the homeostasis of various skin functions and indicated that cannabinoids could also be considered for the treatment and prophylaxis of tumour diseases of the skin. Cannabinoids have been shown to exert their anticarcinogenic effects at different levels of skin cancer progression, such as inhibition of tumour growth, proliferation, invasion and angiogenesis, as well as inducing apoptosis and autophagy. This review provides an insight into the current literature on cannabinoid compounds as potential pharmaceuticals for the treatment of melanoma and squamous cell carcinoma.

## 1. Skin Cancer—Incidence and Current Status of Pharmacotherapy

Skin cancer essentially comprises three tumour categories, namely, cutaneous melanoma, basal cell carcinoma and squamous cell carcinoma. The latter two are grouped under the term non-melanoma skin cancer, which also includes other primary skin neoplasms such as cutaneous lymphoma, adnexal carcinoma, dermatofibrosarcoma protuberans and Merkel cell carcinoma. Therefore, “keratinocyte carcinoma” is the preferred term, combining basal cell carcinomas and cutaneous squamous cell carcinomas because of their common descent from epidermal keratinocytes [1]. Basal cell carcinoma is the most common malignant disease in the world. In the United States of America alone, 4.3 million new cases occur every year. However, many cancer registries do not record this type of skin cancer because of its low mortality rate (for a review, see [2]). Among skin cancers, melanoma is the most aggressive form, with increasing incidence. Survival rates remain poor for higher-stage melanoma, although recent advances in pharmacotherapies such as immunotherapy for metastatic melanoma have improved treatment outcomes. According to the Global Cancer Observatory (GLOBOCAN), in 2020, 324,635 new cases of melanoma of the skin with 57,043 deaths and 1,198,073 cases of non-melanoma of the skin (excluding basal cell carcinoma) with 63,731 deaths have been reported [3]. However, the GLOBOCAN estimates for 2020 do not reflect the impact of severe acute respiratory syndrome coronavirus 2 (SARS-CoV-2) because they were based on extrapolations of cancer data collected in earlier years before the pandemic. For the United States, recent calculations have predicted new melanoma cases in 62,260 men and 43,850 women, of whom 4600 men and 2580 women would be expected to die per year [4]. According to [4], the 5-year survival rate for melanoma was 93%. There is a clear difference between the survival of patients with melanoma that has not yet metastasised or spread regionally at the time of diagnosis (99% 5-year survival rate) and patients with melanoma that has already metastasised at the time of diagnosis (27% 5-year relative survival rate). Regional differences must be taken into account in these epidemiological considerations. For example, non-melanoma skin cancer (excluding basal cell carcinoma) in Australia/New Zealand reaches an age-standardised incidence rate per 100,000 population of 166.2 in men and 110.0 in women, while in various parts of Asia, the corresponding incidences are between 1 and 5 [3]. It is noteworthy that the mortality rate for melanoma significantly decreased by 5.7% annually between 2014 and 2018 [4].

Regarding some previous studies, the treatment of cutaneous melanoma with classical chemotherapeutic agents such as the Dartmouth regimen (dacarbazine, cisplatin, carmustine, and tamoxifen) [5] and with combinations of chemotherapeutic agents such as dacarbazine, platinum compounds, taxanes and vinca alkaloids with interleukin (IL)-2 and interferon (IFN) α-2b [6,7] leads to significant adverse side effects and only moderate progress in terms of patient survival. For this reason, the various options of targeted and immunological therapies such as antibodies against T-lymphocyte-associated protein 4 (CTLA-4, CD152) and programmed death 1 (PD-1) or its programmed death-ligand 1 (PD-L1) are now preferred as first-line therapies for metastatic melanoma. CTLA-4 belongs to the family of CD28/B7 immunoglobulins (Ig) and, as an important checkpoint inhibitor, suppresses T-cell activation, leading to inhibition of tumour defence. The prevention of CTLA-4 binding to its ligand, the B7 protein, by monoclonal antibodies in antigen-presenting cells thus leads to the activation of CD8-positive T cells and supports the body’s own tumour defence. The binding of PD-1 to its ligand, PD-L1, also causes an inhibition of T-cell activation. Subsequently, PD-1 and PD-L1 antibodies have shown promising antitumour activity by suppressing PD-1/ligand binding [8,9,10,11]. Immunotherapy of melanoma preferably consists of the PD-1 blockers nivolumab and pembrolizumab and the CTLA-4 antibody ipilimumab as monotherapies or in combination, the latter of which has advantages for patients with metastatic melanoma. Accordingly, a recent study showed that ipilimumab plus anti-PD-1 therapy (pembrolizumab or nivolumab) was superior to ipilimumab monotherapy in terms of response rate, progression-free survival, and overall survival in patients with metastatic melanoma [12].

Targeted therapies take advantage of the peculiarity of skin cancer as a neoplasm that has the highest number of mutations due to exposure to mutagenic ultraviolet radiation (UV). A single substitution mutation in the v-raf murine sarcoma viral oncogene homologue B (*BRAF*) is found in half of all cases of skin tumours, whereas mutations in the neuroblastoma RAS (rat sarcoma) viral oncogene homologue (*NRAS*) occur in 21% [13]. V600E/K *BRAF* mutations result in activation of the RAS/RAF/mitogen-activated protein/extracellular signal-regulated kinase kinase (MEK)/extracellular-signal regulated kinases (ERK) pathway, leading to constitutive mitogen-activated protein kinase (MAPK) activity in melanoma. In these genotypes of skin cancer, large trials led to approval of vemurafenib [14] and dabrafenib [15] by the U.S. Food and Drug Administration (FDA). Among the targeted molecular therapies that specifically inhibit the BRAF and MEK pathway and that are also approved for the treatment of patients with advanced melanoma, whose tumours have a V600 mutation in the *BRAF* gene, three regimens of inhibitors of this pathway are currently in use, namely, vemurafenib plus cobimetinib, dabrafenib plus trametinib, and encorafenib plus binimetinib (for a review, see [16]).

In addition, cytotoxicity against melanoma can be maximised through the immune response by reintroducing the patient’s own tumour-infiltrating T cells as part of so-called adoptive cell therapy. In this context, clinical response rates of 50% have been observed following the administration of autologous tumour-reactive T cells following lymphocyte-depleting chemotherapy [17,18]. 

Besides surgery, radiotherapy and cryotherapies, systemic 5-fluorouracil as an antimetabolite and topical imiquimod as an imidazoquinolone that stimulates Toll-like receptor 7 are among the pharmacologic interventional therapies for in situ or infiltrating squamous cell carcinoma of the skin [19]. Recently, immunotherapies became the standard of care for patients with cutaneous squamous cell carcinoma with the approval of the anti-PD-1 antibodies cemiplimab and pembrolizumab by the FDA and European Medicines Agency (EMA) (for a review, see [20]). These advances show that, particularly in skin cancer, new successful therapeutic interventions are possible that improve patients’ quality of life and survival. 

Another option in this pharmacological armamentarium could be drugs from the group of cannabinoids. This assumption is based on the now-mature knowledge that the endocannabinoid system is a crucial factor in skin homeostasis and exerts a significant influence on cutaneous pathophysiological processes. Accordingly, the endocannabinoid system has even been referred to as the “c(ut)annabinoid system” in reference to numerous publications on the cutaneous cannabinoid system [21]. 

As a matter of fact, multiple preclinical in vitro and in vivo studies have shown that cannabinoids have potential pharmacotherapeutic beneficial effects in a number of tumour entities. These include effects on various levels of tumour progression, such as inhibition of tumour cell proliferation, induction of apoptosis as well as autophagy processes coupled to it, and likewise inhibition of angiogenesis, invasion and metastasis (for a review, see [22]). The following chapters discuss the preclinical and clinical effects of cannabinoids on melanoma and squamous cell carcinoma found to date in vitro and in vivo. In order to provide a more comprehensive overview of the effects of cannabinoids on squamous cell carcinoma cells, the presentation is not limited to data on cutaneous squamous cell carcinomas but includes reports on squamous cell carcinomas of the oesophagus and other origins. In addition, this review describes effects of cannabinoids on cells derived from Kaposi’s sarcoma. Although cannabinoid receptors are expressed in basal cell carcinomas [23], there are currently no data on probable growth inhibitory effects of cannabinoids on basal cell carcinomas. For this reason, this type of skin cancer was not considered in the present review.

## 2. The Endocannabinoid System

### 2.1. The Endocannabinoid System—A Brief Description of the Components 

The endocannabinoid system includes the endocannabinoid receptors, endogenous agonists and enzymes that synthesise and degrade endocannabinoids. N-arachidonoylethanolamine (anandamide, AEA) and 2-arachidonoylglycerol (2-AG) were the first arachidonic acid derivatives described as endogenous agonists at cannabinoid receptors [24,25]. Later, further lipids were identified as endocannabinoids, such as N-arachidonoyldopamine (NADA) [26]; 2-arachidonoylglycerol ether (2-AGE, noladin ether), a structural ether analogue of 2-AG with higher stability than 2-AG [27]; and O-arachidonoylethanolamine (virodhamine), an ester derivative of arachidonic acid and ethanolamine [28]. In addition, other N-acylethanolamines such as palmitoylethanolamide (PEA), oleoylethanolamide (OEA), stearoylethanolamide (SEA), and linoleoylethanolamide (LEA) have been classified as endocannabinoid-like substances, the latter utilising the bio-synthetic and degradative enzymes of endocannabinoids but not triggering cannabinoid receptor activation (for a review, see [29]).

As early as 1984, Allyn C. Howlett demonstrated that Δ^9^-tetrahydrocannabinol (THC), the main psychoactive compound in *Cannabis sativa* L., reduces cyclic AMP accumulation in neuronal cells via a decrease in adenylate cyclase activity [30]. The receptors discovered later in the early 1990s, which are activated by cannabinoids, are heptahelical pertussis toxin-sensitive G_i/o_ protein-coupled membrane receptors called cannabinoid receptors CB_1_ and CB_2_ [31,32]. With respect to endocannabinoids, AEA was found to be a partial agonist at the CB_1_ receptor, with an affinity comparable to that of THC [33,34]. Further studies revealed that AEA is a weak partial CB_2_ agonist in a mammalian expression system, even antagonising the action of 2-AG at the CB_2_ receptor because of its low intrinsic activity [35]. A study published in the same year, using the human leukaemia cell line HL-60, was able to confirm the weak agonistic effect of AEA at the CB_2_ receptor [36]. On the other hand, 2-AG has been shown to act as a full agonist at the CB_2_ receptor [35,36]. Another paper from this period even demonstrated a superior agonistic effect of 2-AG compared to AEA, 2-AGE, HU-313, R(+)-methanandamide (Met-AEA), and CP 55,940 at the CB_1_ receptor [37]. The authors concluded that 2-AG is more effective than AEA under certain experimental conditions, such as membrane incubation, but degrades faster. Virodhamine, on the other hand, functions as a CB_1_ receptor antagonist and CB_2_ receptor full agonist [28]. According to an early study, NADA has 40-fold selectivity for CB_1_ over CB_2_ and acts as a CB_1_ agonist even more strongly and effectively than AEA in terms of intracellular calcium mobilisation in neuroblastoma cells [26]. Finally, the omega-3 fatty acid ethanolamides N-docosahexaenoylethanolamine (DHEA) and N-eicosapentaenoylethanolamine (EPEA) also act as endocannabinoids, as determined by [^35^S]GTPγS binding assays [38].

Regarding the receptor interaction of phytocannabinoids, THC was found to act as a partial agonist at both cannabinoid receptors with low nanomolar K_i_ values, while the non-psychoactive phytocannabioid cannabidiol (CBD) has only weak cannabinoid receptor affinity with K_i_ values in the micromolar range (for a review, see [39]). Competitive inhibition studies revealed a higher affinity of CBD to CB_2_ than to CB_1_, with K_i_ values of 372.37 nM and 1458.5 nM, respectively [40]. In another in vitro study, CBD was found to act as a non-competitive antagonist of both CB_1_ and CB_2_ receptor agonists [41]. In a recently published molecular dynamics simulation, it was also shown that CBD binding to the N-terminal domain of the CB_1_ receptor leads, in the sense of a negative allosteric regulation, to a change in the orthosteric binding mode of THC [42]. This could provide a plausible explanation for CBD counteracting some of the negative side effects of THC, such as intoxication, sedation, and tachycardia (for a review, see [43]).

The extended endocannabinoid system likewise includes other receptors modulated by cannabinoids, such as transient receptor potential vanilloid 1 (TRPV1), which is activated by AEA [44] and CBD [45]. In these experiments, the latter compound was found to be inactive in affinity binding experiments to CB_1_ and CB_2_ receptors [45]. Moreover, NADA has been shown to increase intracellular calcium concentration via binding to TRPV1 [46]. It was also found that 2-AGE, in addition to its CB_1_ and weak CB_2_ receptor agonist properties [27], acts as a weak TRPV1 agonist in HEK293 cells transfected with a vector stably expressing human TRPV1 [47]. From the group of TRPVs, TRPV2 was also discovered as a target receptor of CBD [48].

The human G protein-coupled receptor 55 (GPR55), which was first isolated and cloned in 1999, is also activated by various cannabinoids in a cell-type- and tissue-dependent manner. Only two studies on this topic are outlined here. In GPR55-transfected cells, EC_50_ values for AEA, 2-AGE, 2-AG, PEA, OEA, abnormal CBD (synthetic regioisomer of CBD) and O-1602 (abnormal CBD analogue) were registered that were lower than the corresponding EC_50_ values in CB_1_ and CB_2_ activity assays, while for CBD an antagonistic effect at GPR55 was demonstrated [49]. In another study, a significant increase in intracellular calcium was registered in GPR55-highly expressing large dorsal root ganglion neurons only by THC, Met-AEA and JWH-015, but not by 2-AG, PEA, virodhamine, CP 55,940, WIN 55,212-2, abnormal CBD or CBD [50]. According to further experiments performed on HEK293 cells in this work, GPR55-induced calcium release appears to involve G_q_ and G_12_ proteins. 

Finally, the proliferator-activated receptor (PPAR) α was reported to be activated by different endocannabinoid-like substances. In this context, OEA [51] and PEA [52] were described as potent PPARα activators. Using reporter assays, another investigation found 30 µM of various endocannabinoids/endocannabinoid-like substances such as OEA, PEA, SEA, LEA, EPEA and DHEA to activate PPARα [53].

Among the cannabinoid compounds, ajulemic acid, a synthetic analogue of the THC metabolite THC-11-oic acid, was first found to bind directly to PPARγ and activate its transcriptional activity [54]. Moreover, THC has been shown to mediate its vasodilatory effect via the activation of PPARγ [55], which was later also confirmed for CBD [56]. Other cannabinoids that act as PPARγ agonists are AEA, 2-AG and NADA as well as the synthetic cannabinoids HU210, WIN 55,212-2 and CP 55,940 (for a review, see [57]).

The synthesis of AEA is mainly carried out by N-acylphosphatidylethanolamine phospholipase D (NAPE-PLD). 2-AG, on the other hand, is synthesised by phospholipase C or by diacylglycerol lipase α and β (for a review, see [58]). The degradation of AEA in the cell is achieved by the enzyme fatty acid amide hydrolase (FAAH) [59], while 2-AG is mainly metabolised by monoacylglycerol lipase (MAGL) or α/β-hydrolase domain-containing (ABHD)-6 and -12 [60]. In addition, AEA was found to be oxidised by cyclooxygenase (COX)-2, leading to prostaglandin (PG) E_2_ ethanolamide [61]. Regarding the oxidation of 2-AG, an early study showed that COX-2 also converts 2-AG, in this case to PGH_2_ glycerol ester [62]. Using cultured macrophages, the authors of the latter study also found that PGH_2_ glycerol ester is subsequently converted to PGE_2_ glycerol ester by PGE synthase and to PGD_2_ glycerol ester by PGD synthase. Later, the same group discovered that AEA and 2-AG can be further metabolised by thromboxane and prostacyclin (PGI) synthase to thromboxane and PGI ethanolamides and glycerol esters, respectively [63]. Accordingly, it can be assumed that the entire range of eicosanoids can also be formed as ethanolamides or glycerol esters from the precursors AEA and 2-AG. In addition, the resulting arachidonic acid residue from the conversion of AEA and 2-AG by FAAH and MAGL is converted to PGs by COX.

**Figure 1:**
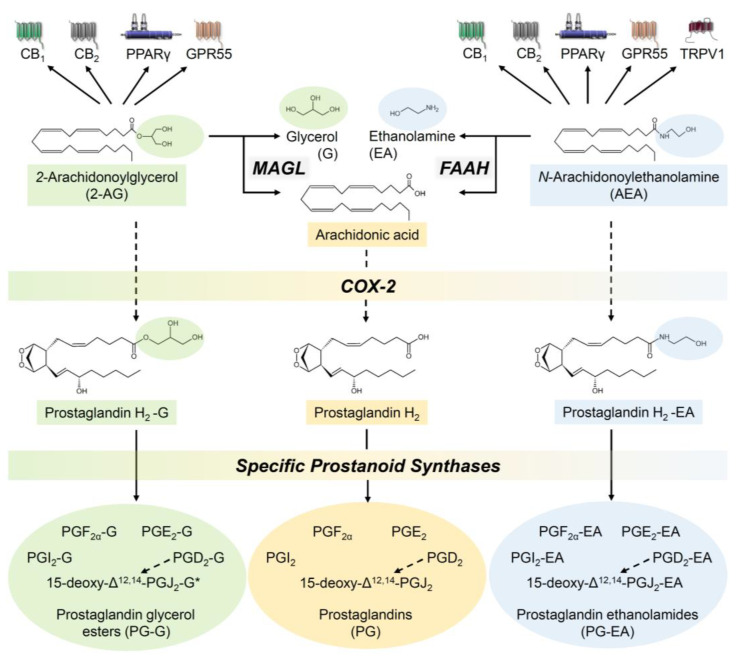
Degradation of AEA and 2-AG by FAAH, MAGL and COX-2 to analytically detectable or putative (*) metabolites. The arrows indicate activations or inductions. Dashed arrows show that various intermediate steps in the metabolism lead to the next parameter. All abbreviations are given in the text.

Figure 1 provides an overview of the described pathways of endocannabinoid degradation. Endocannabinoid-derived thromboxanes were omitted, as these represent rather unlikely biologically significant mediators [63].

### 2.2. The Endocannabinoid System in the Skin

The presence of the endocannabinoid system in skin tissue was demonstrated in the mid-1990s by the discovery of the endocannabinoid AEA in rat skin, where it was found in concentrations similar to those in the rat brain [64]. Later, endocannabinoids were shown to cause tonic activation of local cannabinoid receptors in rat skin [65]. Regarding the distribution of cannabinoid receptors in the human skin, both subtypes were found in small afferent peptidergic nerves, but also in mast cells, macrophages, epidermal keratinocytes and epithelial cells of hair follicles, sebocytes and eccrine sweat glands [66]. In addition, TRPV subtypes 1–4 have been detected in nerve endings, eccrine sweat glands, hair follicles and the dermis of the skin, as recently reviewed [67]. Among the PPARs as further cannabinoid-activated receptors, the expression of the PPARγ subtype in rat preputial sebocytes was described foremost [68]. Meanwhile, numerous publications indicate that PPARα, δ or γ activation is crucial for epidermal and keratinocyte functions and promotes skin integrity (for a review, see [69]). 

The great importance of cannabinoid receptors for the homeostasis of the skin was clarified in a comprehensive study that demonstrated an involvement of these receptors in the pathogenesis of allergic contact dermatitis [70]. The authors of the latter study found that CB_1_/CB_2_ receptor double knockout mice showed increased allergic inflammation, while conversely, FAAH-deficient mice with correspondingly higher AEA levels exhibited fewer allergic skin reactions. In line with this, numerous studies on the role of endocannabinoids in various skin functions have led to these compounds now being considered important neuroendocrine regulators in the maintenance of skin homeostasis [71]. Accordingly, the endocannabinoid system has been implicated in several physiological processes of the skin, e.g., melanogenesis [72], regenerative capabilities [73,74] and modulation of cutaneous immune cells [75]. Currently, a number of studies are investigating cannabinoids as a treatment option for various skin conditions, such as atopic dermatitis, systemic sclerosis, dermatomyositis, epidermolysis bullosa and pyoderma gangrenosum.

### 2.3. Endocannabinoid System in Melanoma and Squamous Cell Carcinoma Tissue

Recently, several studies have investigated possible regulations of cannabinoid receptors and other elements of the endocannabinoid system in skin cancer tissues, as well as a possible link between these factors and skin tissue malignancy. In this context, one study showed that the CB_2_ receptor is upregulated in melanoma tissue [76]. Other efforts to study the effect of cannabinoids and the endocannabinoid system on cancer progression have included analyses of cannabinoid receptor expression levels in cancer tissue and their association with malignancy and survival in patients with squamous cell carcinoma. For example, CB_1_ receptor-positive squamous cell carcinomas of the oesophagus were associated with poorer patient survival compared with patients with CB_1_ receptor-negative tissue [77]. Another study showed that strong CB_2_ receptor immunoreactivity in biopsies from patients with squamous cell carcinoma of the head and neck was significantly associated with a reduced disease-specific survival rate [78]. In contrast to these findings, increased expression of cannabinoid receptors was found to be associated with significantly longer overall survival and disease-free survival in patients with mobile squamous cell carcinoma of the tongue [79]. This study also uncovered age and sex differences, with increased CB_2_ receptor expression and concurrent CB_1_/CB_2_ receptor expression occurring more frequently in female than male patients. An increase in cannabinoid receptors was also observed more frequently in older than in younger patients. Therefore, it is difficult to make a statement about whether the regulation of cannabinoid receptors in tumour tissue in melanoma or squamous cell carcinoma is predictive of disease progression or even of the functional role of these receptors in the pathogenesis of these cancers.

Regarding the regulation of other elements of the endocannabinoid system in skin cancer, one study found upregulation of MAGL in melanoma tissue, with increased expression associated with higher tumour aggressiveness [80].

## 3. Inhibition of Tumour Growth of Melanomas and Squamous Cell Carcinomas by Cannabinoids

### 3.1. Antiproliferative and Proapoptotic Effects of Cannabinoids on Melanoma

There have been numerous investigations into the antiproliferative and proapoptotic effects of cannabinoids on human melanoma cancer cells expressing CB_1_ and CB_2_ receptors. In 2006, Blázquez et al. showed that activation of CB_1_ and CB_2_ leads to a reduction in growth, proliferation, and metastasis of melanoma cells, as well as neovascularisation of melanoma xenografts [81]. Part of this cannabinoid-induced antiproliferative activity was due to modulation of cell cycle checkpoints. Thus, WIN 55,212-2 induced melanoma cell cycle arrest through inhibition of protein kinase B (Akt) and hypophosphorylation of retinoblastoma-associated protein (Rb) [81]. In addition, this study found that 0.1 µM WIN 55,212-2 or 1 µM THC decreased the viability of melanoma cell lines B16 and A375, which was reversed in the presence of CB_1_ and CB_2_ antagonists. A similar decrease in viability by cannabinoids could not be detected in non-transformed melanocytic cell lines. The authors also reported a pronounced inhibitory effect of WIN 55,212-2 or JWH-133 at 50 µg per day on the growth of xenografts generated by injection of B16 mouse melanoma cells into C57BL/6 mice. In another work, WIN 55,212-2 was shown to have an antiproliferative effect on the metastatic skin melanoma cell line SK-MEL28 and the non-metastatic ocular uveal melanoma cell line OCM-1 via a receptor-independent pathway involving lipid rafts [82]. Here, CB_1_ and CB_2_ antagonists did not rescue melanoma cells from cannabinoid-induced cell death, whereas disruption of lipid rafts by methyl-β-cyclodextrin protected cancer cells. A recent study tested the effect of CBD on malignant melanoma cell growth and metastasis in a mouse model in which B16F10 mouse melanoma cells were injected into C57BL/6 mice [83]. Compared with the control group, a significant decrease in tumour size was observed in the CBD-treated mice. The survival time of the CBD-treated animals was significantly longer than that of animals in the control group, with the cisplatin-treated comparison group having the longest survival time. On the other hand, quality of life and physical performance were better in CBD-treated mice [83]. A further study found that CBD oil significantly inhibited cell growth of B16 melanoma cells in vitro [84]. However, the proapoptotic mechanism of CBD in melanoma cells is not yet known.

As for combinations with radiation treatments, a standardised *Cannabis sativa* extract alone or in combination with a single dose of radiation significantly inhibited melanoma cell viability and proliferation in vitro in a concentration-dependent manner. Remarkably, this inhibition of melanoma cell viability was accompanied by an increase in necrosis but not apoptosis [85].

A number of publications further found endocannabinoids and inhibitors of endocannabinoid-degrading enzymes to influence melanoma growth. Accordingly, antitumour activity of AEA on A375 melanoma cells was reported in a study showing concentration-dependent cytotoxicity of AEA in association with a caspase-dependent apoptotic signalling pathway [86]. Here, cytotoxicity of AEA was enhanced by inhibition of FAAH and attenuated by inhibition of COX-2 or lipoxygenase (LOX). Blocking CB_1_ receptors partially reduced the cytotoxicity of AEA. In contrast, methyl-β-cyclodextrin, tested at a concentration sufficient to destroy membrane lipid rafts without affecting viability, completely reversed cytotoxicity by AEA and by the GPR55 agonist O-1602, suggesting a possible role of lipid rafts and GRP55 in the viability-impairing effect of AEA [86]. Another investigation found that AEA, PEA and 2-AG reduced the viability of B16 mouse melanoma cells [87]. The study mainly focused on the antiproliferative effect of PEA, which was further enhanced by the inhibition of FAAH and thus the inhibition of PEA hydrolysis. This effect was also confirmed in a xenograft model in mice, where treatment with PEA alone and even more so in combination with the FAAH inhibitor URB597 resulted in profound inhibition of melanoma growth [87]. 

In addition, MAGL has been shown to be a factor that decisively influences the malignancy of melanoma cells. One study showed that the melanoma cell line C8161, in which MAGL was permanently downregulated by transfection with a small hairpin (sh) RNA, exhibited reduced viability. Interestingly, the authors could not confirm inhibition of cell survival when using the MAGL inhibitor JZL184. However, both MAGL shRNA and JZL184 inhibited xenograft growth in vivo, with the effect of MAGL shRNA being abolished by a high-fat diet [88]. Consistent with a MAGL-dependent tumour progression, the same study also found that overexpression of MAGL in the metastatic uveal melanoma cell line MUM2C induced enhanced tumour growth in vivo. 

Finally, PGD_2_ ethanolamide [89] and 15-deoxy-Δ^12,14^-PGJ_2_ ethanolamide [90], both products of COX-2-dependent metabolism of AEA (Figure 1), were described as inducers of apoptotic cell death of B16F10 melanoma cells, with the latter compound also reducing B16F10 solid tumour growth in C57BL/6 mice [90].

It is also noteworthy that cannabinoid receptor activation is not a process that has been uniformly described as growth inhibitory. For example, in A375 melanoma cells, the inverse CB_1_ agonist AM-251 has been associated in vitro with arrest of the G2-M cell cycle, inhibition of the expression of anti-apoptotic proteins such as B-cell lymphoma 2 (BCL2) and survivin, and increased expression of the proapoptotic Bcl-2-associated X protein (BAX) [91]. Consistent with the expected mechanism of AM-251 as an antagonist/inverse agonist at the G_i/o_ protein-coupled CB_1_ receptor, a 40% increase in basal cAMP levels was observed after treatment of A375 melanoma cells with AM-251, although its causal involvement in cell cycle arrest was not further investigated [91]. In another study, however, silencing of CB_1_ using shRNAs, led to a slowing in the cell cycle in the G1-S phase, which was accompanied by reduced activation of protein kinase B (Akt) and ERK, suggesting a possible function of the CB_1_ receptor as a tumour-promoting signal in human skin melanomas [92]. In a further study, AEA, 2-methyl-2′-fluoro-anandamide (Met-F-AEA) and arachidonyl-2-chloroethylamide (ACEA) were shown to inhibit the proliferation of the melanoma cell lines HT168-M1, WM35 and HT199 [93]. Paradoxically, this study also found inhibition of proliferation by the CB_1_ receptor antagonist/inverse agonist AM-251.

It can be assumed that the mode of action of cannabinoids under certain conditions can induce concentration-dependent opposite effects with low, proliferatively effective concentrations and high antiproliferative and proapoptotic concentrations. Thus, Met-AEA and other cannabinoids, such as AEA, the CB_1_ agonist ACEA and 2-AG, induced melanogenesis via CB_1_ activation up to a concentration of 3 µM, whereas apoptosis of primary normal human epidermal melanocytes occurred via activation of TRPV1 at high concentrations of Met-AEA (≥4 µM) [94].

**Figure 2:**
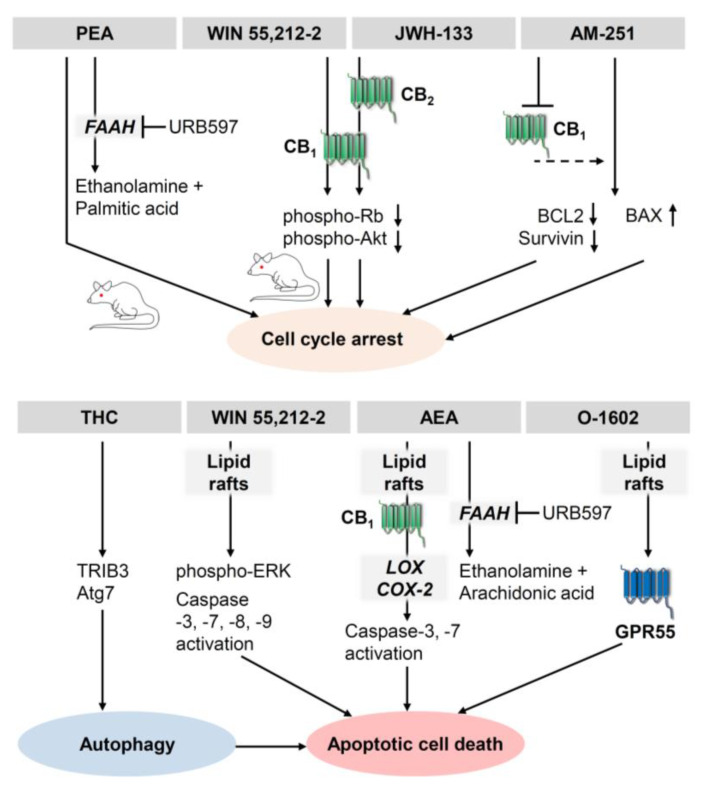
Mechanisms of the growth inhibitory (**upper** panel) and proapoptotic (**lower** panel) effects of cannabinoids on melanoma cells demonstrated so far. Where a mouse is shown, the data were confirmed by in vivo experiments. The figure does not include experimental data from publications in which no experiments on the intracellular mechanism of action of cannabinoids on melanoma cells were performed. The arrows indicate the sequential causal relationships of activations, inductions, downregulation and inhibitions triggered by the indicated substances. In the cases where URB597 is listed with an inhibitory arrow directed at FAAH, URB597 resulted in an enhancement of the endocannabinoid effect shown. The dashed arrow indicates that a causal relationship between CB_1_ receptor inactivation and the subsequent signalling pathway has not been shown. All abbreviations are given in the text.

Figure 2 gives an overview of the antiproliferative and proapoptotic effects of different cannabinoids on melanoma cells.

### 3.2. Antiproliferative and Proapoptotic Effects of Cannabinoids on Squamous Cell Carcinomas

As for melanomas, there are several studies on possible antiproliferative and proapoptotic effects of (endo)cannabinoids on squamous cell carcinomas. In this context, the finding published by Casanova et al. in 2003 [23] that the synthetic cannabinoid WIN 55,212-2 and the selective CB_2_ agonist JWH-133 induce apoptosis in a mouse spindle cell squamous cell carcinoma cell line (PDVC 57B cells) in vitro and reduce tumour growth in a murine xenograft model represents the first ever report of anticarcinogenic cannabinoid effects in skin cancer.

It was later shown that cannabinoids in squamous cell carcinomas lead to the death of the cancer cells not only through their effect on the cannabinoid receptors, but also through other mechanisms of action. In this context, the fact that most non-melanoma skin cancers as well as other epithelial tumours overexpress COX-2 plays an important role. According to van Dross et al., treatment with AEA in the murine COX-2-overexpressing keratinocyte squamous cell carcinoma cell line JWF2 led to cell death due to the metabolism of AEA to cytotoxic PGD_2_ and PGD_2_-ethanolamide as well as PGJ_2_ and 15-deoxy-Δ^12,14^-PGJ_2_ [95]. The authors did not observe any cytotoxic effect of AEA in the wild-type HaCaT keratinocyte cell line with low COX-2 expression. However, the effects were detected in cells after transfection of a COX-2-containing vector, which led to COX-2 overexpression in HaCaT cells. In another investigation using JWF2 cells, the authors also found that AEA-induced apoptosis could be inhibited by the antioxidant N-acetylcysteine but not by cannabinoid receptor or TRPV1 antagonists, suggesting receptor-independent involvement of reactive oxygen species (ROS) in AEA-induced apoptosis [96]. Furthermore, the latter work showed that FAAH inhibitors further enhanced AEA-induced synthesis of J-series PGs and thus apoptosis. A follow-up investigation by the same group additionally revealed that AEA triggers the activation of PKR-like endoplasmic reticulum kinase (PERK) and its downstream target, eukaryotic initiation factor 2α (eIF-2α), in JWF2 cells [97]. This was accompanied by a transient upregulation of serine/threonine protein kinase/endoribonuclease-requiring enzyme 1 (IRE1) phosphorylation and subsequent downregulation of unspliced (inactive) X-box-binding protein 1 (XBP-1), which serves as a marker of phospho-IRE1 endoribonuclease activity. These regulations all point to signalling pathways associated with excessive endoplasmic reticulum stress. As a characteristic endoplasmic reticulum-associated apoptosis target, AEA induced C/EBP homologous protein-10 (CHOP10) and caused downstream activation of caspase-12 and caspase-3 in tumorigenic keratinocytes overexpressing COX-2. In AEA-treated JWF2 cells, liquid chromatography–tandem mass spectrometry analysis revealed increases in PGJ_2_ ethanolamide, Δ^12^-PGJ_2_ ethanolamide and 15-deoxy-Δ^12,14^-PGJ_2_ ethanolamide, metabolites of AEA formed via COX-2 that cause stress in the endoplasmic reticulum, finally leading to apoptotic cell death [97]. Finally, the apoptotic effect induced by enzymatic products of COX-2-dependent metabolism of AEA in JWF2 cells was confirmed with the finding that PGD_2_ ethanolamide leads to apoptotic cell death of skin cancer cells via both oxidative and endoplasmic reticulum stress [89]. At the mechanistic level, suppression of the glutathione and thioredoxin systems has been shown to cause oxidative stress without the involvement of D-prostaglandin (DP) receptors and PPARγ. 

**Figure 3:**
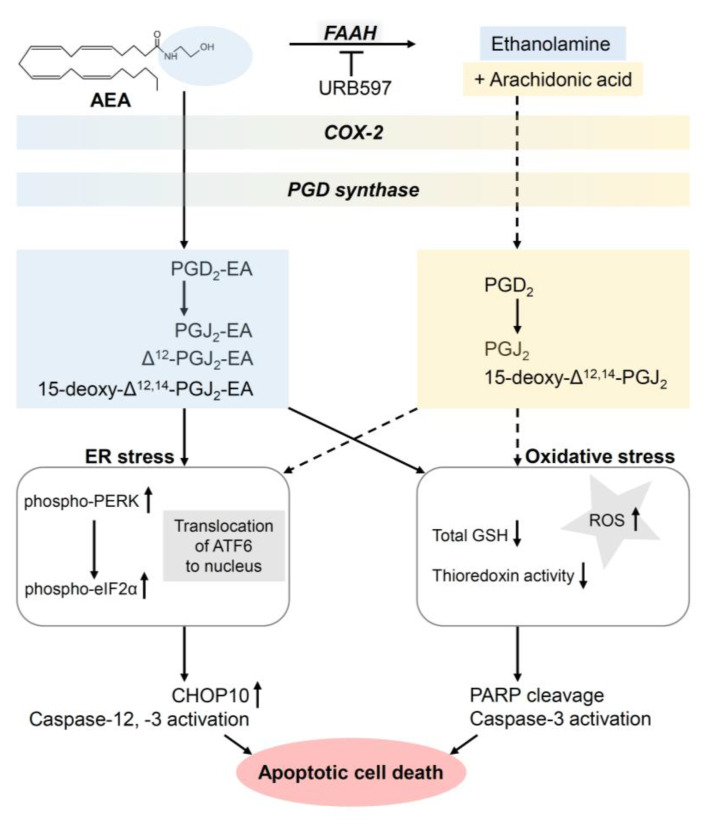
Proapoptotic effect of AEA and its metabolites formed via COX-2 and PGD synthase on tumorigenic keratinocytes. The arrows indicate the sequential activations and inductions triggered by the indicated substances. In the case where URB597 is listed with an inhibitory arrow directed at FAAH, URB597 resulted in an enhancement of the AEA effect shown. The dashed arrows indicate possible additional effects of AEA after degradation to arachidonic acid. All abbreviations are given in the text.

Figure 3 provides an overview of the mechanisms of the proapoptotic effect of AEA on tumorigenic keratinocytes.

Furthermore, AEA, but not 2-AG, was found to exert an anticancer effect on head and neck squamous cell carcinoma cell lines (SNU-1041, SNU-1066 and PCI-1) via a receptor-independent mechanism mediated by an increase in ROS [98]. It is noteworthy that in the latter study, no involvement of COX-2 in AEA-induced apoptosis was detected. In line with this finding, the authors were also unable to detect any cytotoxic effect of the main substrate of PG synthesis, arachidonic acid. One year later, the same group published the effects and mechanisms of action of further ethanolamides derived from polyunsaturated fatty acids (PUFAs), which are structurally similar to AEA, on head and neck squamous cell carcinoma cell lines [99]. This demonstrated an antiproliferative effect of DHEA and N-arachidonoyl-L-alanine (NALA) that was independent of CB_1_ receptor and TRPV1 activation and instead partially abrogated by antioxidants and 5-LOX inhibition. Further experiments showed that in addition to the growth inhibitory effects, the ROS-increasing and Akt phosphorylation-inhibiting effects of the PUFAs tested were reduced by inhibition of 5-LOX, suggesting that ROS generated by the 5-LOX pathway mediate the anticancer effects of DHEA and NALA [99]. 

Regarding the anticancer effects of phytocannabinoids, CBD was demonstrated to significantly reduce cell viability of the human tongue carcinoma cell line SCC-25 and induce apoptosis [100]. In this study, however, CBD was found to have an effect exclusively at relatively high concentrations (≥30 µM). In another study, CBD showed a synergistic inhibitory action with anticancer drugs such as cisplatin, paclitaxel and 5-fluorouracil on the viability and colony-forming activity of head and neck squamous cell carcinoma cells [101]. Finally, a recent study revealed an antiproliferative effect of AEA and 2-AG on the human laryngeal cancer cell line HEp-2 [102].

To determine the contribution of GPR55 as another cannabinoid-driven target to squamous cell carcinoma carcinogenesis, a study was conducted using GPR55-deficient mice. Here, GPR55 was shown to increase the number of papillomas and carcinomas in a mouse skin carcinogenesis model. Using the murine transformed mouse keratinocyte cell line PDV, the authors also found that cells with stable suppression of GPR55 had lower viability, colony formation and invasion in vitro, and lower xenograft tumour growth in vivo [103]. However, the involvement of endocannabinoids in carcinogenesis and the druggability of GPR55 as a target for the treatment of squamous cell carcinoma were not investigated in this study, as this would require a pharmacological experimental approach with GPR55-inhibiting cannabinoids.

As with the effects of cannabinoid receptor-associated signalling pathways in melanoma cells, antagonisation rather than activation of cannabinoid receptors has been shown to mediate anticarcinogenic effects in squamous cell carcinoma cells. Accordingly, using the human papillomavirus (HPV)-positive squamous cell carcinoma cell lines UD-SCC-2, UPCI-SCC-090, UM-SCC-47 and 93VU147T, it has been reported that cannabinoid receptor silencing inhibits the proliferation of these cancer cells [104]. In the latter study, the CB_1_ agonist ACEA, the CB_2_ agonist HU308 and THC were found to increase proliferation in three out of five different HPV-positive head and neck squamous cell carcinoma cell lines at concentrations up to 1 µM after an incubation period of three days. ACEA and HU308 left proliferation virtually unchanged in the other two cell lines, while THC inhibited proliferation in one cell line and had no effect in another cell line. ACEA and HU308, at concentrations of 10 µM each, resulted in additional inhibition of cell growth in another of the five cell lines (UM-SCC-47). The selective CB_1_ receptor antagonist rimonabant and the CB_2_ receptor antagonist SR144528 each inhibited proliferation in three of these five cell lines and had no effect on cell growth in the other two cell lines. The authors also confirmed the trend of these results in vivo with rimonabant, SR144528 and suppression of CB_1_ and CB_2_ by shRNA transfection, each of which reduced the growth of xenografts of UD-SCC-2 cells in nude mice. The administration of THC accelerated tumour growth accordingly [104]. Hijiya et al. also published results on squamous cell carcinomas of the oesophagus, which showed a strong correlation between higher CB_1_ receptor expression and the degree of metastasis to lymph nodes. Consistent with this notion, the authors found that the inverse CB_1_ agonist AM-251 significantly suppressed the proliferation of several oesophageal cancer cell lines [77].

### 3.3. Antiproliferative Effects of Cannabinoids on Kaposi Sarcoma and Cutanous Lymphoma Cells

Finally, it should be mentioned that with regard to cannabinoid effects, other skin cancer types have been investigated in addition to melanoma and squamous cell carcinoma cells. For example, cannabinoids have been successfully tested preclinically for their anticarcinogenic effects on cells of Kaposi’s sarcoma, a tumour of endothelial origin with multiple lesions of the skin, the etiological agent of which is a Kaposi’s sarcoma-associated herpes virus (KSHV), also known as human herpesvirus 8 (HHV-8). In this context, CBD was shown to inhibit proliferation and induce apoptosis in KSHV-infected human dermal microvascular endothelial cells [105]. The opposite effect was reported several years earlier for THC, which increased viral G protein-coupled KSHV receptor expression and viral load in human skin microvascular endothelial cells. Consequently, THC promoted the transformation of infected endothelial cells, as evidenced by colony-forming properties that were markedly increased at 0.01 µM THC [106]. Using the human Kaposi’s sarcoma cell line KS-IMM, further studies showed that WIN 55,212-2 at a concentration of 2 µM inhibited proliferation associated with activation of caspase-3 and -6 and MAPKs, whereas ACEA and JWH-133 showed no comparable effects at up to 5 µM. The antiproliferative effect of WIN 55,212-2 was partially restored in the presence of a CB_2_ receptor antagonist [107]. In a recently published study, different extract fractions of cannabis were tested for their anticarcinogenic effect in the cutaneous T-cell lymphoma (CTCL) cell lines, My-La and HuT-78, but also in peripheral blood lymphocytes from patients with the leukaemic stage of CTCL, also known as Sézary, with characteristic Sézary T cells [108]. Thereby, the researchers were able to identify crude extract fractions that induced cell cycle arrest in the aforementioned cell types, suggesting that cannabinoids could serve as a therapeutic option for cutaneous lymphomas. 

### 3.4. Effect of Cannabinoids on Autophagy of Melanoma and Squamous Cell Carcinoma Cells

As a further mode of action, treatment of melanoma cells with cannabinoids resulted in loss of cell viability due to activation of autophagy in several publications. The induction of autophagy in the melanoma cell lines HL-1, A375 and SK-MEL-28 after treatment with THC and the resulting apoptosis was prevented by simultaneous treatment with chloroquine, a classical inhibitor of autophagy that acts by changing the acidic content of the lysosomes [109]. THC-induced autophagy was associated with an increase in LC3-II, a characteristic feature of early-stage autophagy. Silencing of autophagy 7 (Atg 7), another marker of autophagy, restored loss of viability in response to THC. According to [109], silencing of tribbles pseudokinase 3 (TRIB3) preserved melanoma cells from THC-induced cell death, whereas silencing of Beclin-1 and the autophagy and Beclin-1 regulator 1 (Ambra1) virtually did not alter THC-induced autophagy-dependent apoptosis. Moreover, THC treatment of mice with *BRAF* wild-type melanoma xenografts generated with CHL-1 melanoma cells or treatment with a combination of THC and CBD resulted in a stronger reduction in cancer growth than treatment with temozolomide [109].

Triggering of autophagy processes by cannabinoids in squamous cell carcinoma cells was demonstrated only in those of the head and neck treated with CBD [101]. In the latter investigation, the cytotoxic effect of CBD was partially abolished by chloroquine in the squamous cell carcinoma cell line FaDu; the laryngeal carcinoma cell line Hep2, which remarkably was shown to be a derivative of the cervical carcinoma cell line HeLa [110]; and the tongue squamous cell carcinoma cell line SCC15.

## 4. Inhibition of Skin Cancer Angiogenesis by Cannabinoids

Tumours require an adequate supply of oxygen and nutrients to grow. To meet this need, tumours are able to promote the formation of new blood vessels, a condition that represents a potential target for therapeutic agents. In xenografts of the epidermal tumour cell line PDV C57B, derived from spindle cell squamous cell carcinoma of mouse skin, Casanova et al. found an impairment of tumour vascularisation and decreases in several growth factors associated with angiogenesis, such as vascular epithelial growth factor (VEGF), placenta-derived growth factor (PlGF) and angiopoietin-2, after treatment of nude mice with WIN 55,212-2 and JWH-133. In addition, both cannabinoids reduced epidermal growth factor (EGF) receptor activation in tissues derived from PDV C57B xenografts. [23]. An anti-angiogenic effect was later confirmed in xenografts of B16 melanoma cells in C57BL/6 mice by demonstrating a reduction in the angiogenesis marker CD31 after treatment with WIN 55,212-2 and JWH-133 [81]. With regard to the effect of endocannabinoid-like compounds on tumour angiogenesis, it was demonstrated using CD31 immunostaining of xenografts with B16 cells in C57BL/6 mice and tube formation assays with human umbilical vein endothelial cells (HUVEC) that PEA alone or in combination with the FAAH inhibitor URB597 leaves the neovascularisation of the xenografts and the angiogenic capacities of HUVEC virtually unchanged, although the compounds strongly inhibit xenograft growth [87].

## 5. Inhibition of Melanoma and Squamous Cell Carcinoma Motility, Invasion, and Metastasis by Cannabinoids

### 5.1. Inhibition of Melanoma Cell Motility, Invasion, and Metastasis by Cannabinoids

Several publications have indicated that cannabinoids inhibit melanoma cell motility, invasion, and metastasis. In this context, activation of the CB_2_ receptor with JWH-133 was shown to reduce melanoma cell attachment to brain endothelial cells and transendothelial migration of melanoma cells [111]. These in vitro data suggest that activation of the CB_2_ receptor can reduce the transmigration of melanoma cells across the blood–brain barrier in the context of the formation of parenchymal brain metastases. Further in vitro work revealed that migration of B16F10 melanoma cells was reduced by treatment with a standardised cannabis extract [112].

In vivo studies in mice found that treatment with WIN 55,212-2 reduced the number of metastatic nodules in the lungs and livers of immunocompetent C57BL/6 and immunodeficient nude mice injected with B16 melanoma cells [81]. In another investigation using a model of splenic liver metastasis with immunodeficient mice, in which lymph node-derived metastatic human amelanotic melanoma cells of the lineage HT168-M1 were xenotransplanted by injection into the spleen, it was demonstrated that ACEA reduced liver metastasis compared with vehicle controls [93]. Notably, AEA had no comparable effect on liver metastasis in the latter study but exerted antimigratory properties on HT168-M1 cells in vitro. In a further publication, a possible link between endocannabinoid levels and the number of metastases spreading to the lungs, spleen, peritoneum and lymph nodes was investigated using a syngeneic melanoma model in mice [113]. Here, plasma levels of AEA, OEA, and PEA were significantly lower in C57BL/6J mice xenotransplanted with a local paw tumour containing B16 mouse melanoma cells than in control mice, while plasma 2-AG levels were increased, but without reaching statistical significance. Following intravenous injection of B16 tumour cells simulating a metastatic process, plasma endocannabinoid concentrations increased transiently, with 2-AG and 1-AG showing a long-lasting increase, suggesting that endocannabinoids may be related to melanoma metastasis in vivo. As OEA showed the most significant initial increase in the metastasis model, the potential antimigratory properties of OEA were further investigated. OEA concentrations of ≥2 µM were found to inhibit migration, whereas OEA at lower concentrations promoted B16 tumour cell migration [113].

The results of another study suggested that MAGL may play an important role in the malignancy of melanomas [88]. The melanoma line C8161 showed attenuated migration and invasion when MAGL was knocked down by specific shRNA. Conversely, stable MAGL overexpression in the metastatic uveal melanoma cell line MUM2C resulted in enhanced migration and invasion compared with control cells, i.e., cells expressing an empty vector or a catalytically inactive version of MAGL. In mechanistic studies performed on C8161 melanoma cells, the addition of free fatty acids, but not CB_1_ or CB_2_ receptor antagonists, restored migration suppressed by MAGL shRNA, suggesting that the mechanism of MAGL-mediated cancer aggressiveness in these cells is mediated by free fatty acids rather than by reduction in the MAGL substrate 2-AG. Consistent with this, the addition of free fatty acids also abolished the migration inhibition induced by JZL184 in MAGL-overexpressing MUM2C melanoma cells [88].

### 5.2. Inhibition of Squamous Cell Carcinoma Motility, Invasion, and Metastasis by Cannabinoids

Data on a possible impact of cannabinoids on squamous cell carcinoma migration, invasion, and metastasis are limited to a few studies. In one study, CBD was reported to reduce invasion of squamous cell carcinoma of the oral tongue cell line SCC15 in a concentration-dependent manner at low micromolar concentrations (4–8 µM) [101]. Remarkably, this study showed that CBD (5 mg/kg) by intraperitoneal administration three times per week reduced the tumour area that developed after injection of the hypopharyngeal cell carcinoma cell line FaDu into the tongue of nude mice. Conversely, another report found that the inverse CB_1_ receptor agonist AM-251 inhibited the invasion of the oesophageal cancer cell lines TE-1, TE-8 and T-Tn [77]. Consistent with the latter notion, ACEA, HU308 and THC at a concentration of 1 µM each were found to enhance migration of the HPV-positive squamous cell carcinoma cell lines UD-SCC-2, UPCI-SCC-090, UM-SCC-104 (ACEA and HU308 only) and UM-SCC-47 (THC only), while the CB_1_ antagonist rimonabant and the CB_2_ antagonist SR144528 at 1 µM each resulted in inhibition of migration of UD-SCC-2, UM-SCC-47 (rimonabant only), UPCI-SCC-090 (SR144528 only) and 93VU147T cells [104]. 

## 6. Impact of Cannabinoid Compounds on Skin Tumour Immune Response

It is known that cannabinoid compounds influence the functional activities of immune cells. Therefore, the role of cannabinoids in the immune defence of tumours has been critically discussed. About 30 years ago, it was already proven that THC and 11-hydroxy-THC suppress the cytolytic activity of cultured natural killer cells [114,115]. Later, it was described that phytocannabinoids suppress signal transduction triggered by T-cell receptors [116,117]. In contrast, however, one study concluded that the cannabinoids CBD, THC and Met-AEA induce lymphokine-activated killer cells to increase disintegration of lung tumour cells [118]. Despite this, the effect of natural killer cells challenged by cannabinoids on skin cancer cells is not yet clear. 

To further deepen the knowledge about the effect of cannabinoids on the immunological tumour defence of melanoma cells, the effect of systemically administered THC on the growth of the melanoma cell line HCmel12 was investigated in a mouse model [119]. Flow cytometric analysis of melanoma xenografts showed a decrease in CD45-positive immune cells and thus reductions in the numbers of macrophages and neutrophils in the cancer tissue after treatment with THC. In this report, no differences were demonstrated between wild-type and cannabinoid receptor double knockout mice in different animal models. These models included a fibrosarcoma model, in which 3-methylcholanthrene was applied once; a skin papilloma model, in which mice were inoculated subcutaneously with 7,12-dimethylbenz[a]anthracene (DMBA) once, which was followed by repeated application of 12-O-tetradecanoylphorbol-13-acetate (TPA); and a melanoma mouse model, in which cannabinoid receptor double knockout mice were crossed with melanoma-prone Hgf-Cdk4^R24C^ mice, with melanoma growth induced by a single epicutaneous application of DMBA. In view of these data, the role of cannabinoid receptors in chemically induced skin cancer appears to be of secondary importance. However, as a consequence of an antagonistic effect on the proinflammatory microenvironment, THC inhibited tumour growth of HCmel12 melanoma xenografts, which was associated with reduced infiltration of protumorigenic myeloid immune cells into the tumour microenvironment [119]. Interestingly, an early study that already considered the immune system aspect of the antimetastatic effects of cannabinoids on melanoma cells showed that metastasis to the lung and liver was inhibited by WIN 55,212-2 in immunocompetent mice to a comparable extent as in immunocompromised mice [81]. In summary, the current data do not allow any consistent conclusions on the extent to which cannabinoids affect the tumour-inhibiting immune system in skin cancer.

## 7. Cannabinoids and Skin Cancer Prevention

The results of some experimental setups demonstrating a chemopreventive effect of the test substances used give hope that cannabinoids could possibly exert a skin tumour-preventive effect. The successful preclinical testing of cannabinoids in inflammatory skin diseases supports this hypothesis insofar as inflammatory processes are associated with tumour-promoting effects. Accordingly, the non-psychoactive cannabinoids cannabichromene (CBC), cannabidivarin (CBDV), cannabigerol (CBG), cannabigerovarin (CBGV) and Δ^9^-tetrahydrocannabivarin (THCV) were found to downregulate lipopolysaccharide-induced release of the proinflammatory cytokines IL-1α, IL-1β, IL-6, IL-8 and tumour necrosis factor (TNF)-α in sebocytes [120]. Other cannabinoids with anti-inflammatory effects on skin cells are S-777469 and lenabasum. S-777469, a 3-carbamoyl-2-pyridone system with carboxylic acid at the 3-position [121], is a novel selective CB_2_ receptor agonist that showed an inhibitory effect on 1-fluoro-2,4-dinitrobenzene (DNFB)-induced ear inflammation in Balb/c mice [122]. Lenabasum, a 9-carbon 1,1-dimethylheptyl side-chain analogue of THC-11-oic acid (also known as JBT-101 or ajulemic acid), unlike the classic non-steroidal anti-inflammatory drugs, does not block inflammatory processes but instead accelerates the resolution of inflammation once it has occurred. Lenabasum is currently being clinically tested as a novel anti-inflammatory and curative drug for various diseases such as dermatomyositis, systemic sclerosis, cystic fibrosis, and systemic lupus erythematosus. According to a mechanism of action proposed by Burstein [123], enzymes of the eicosanoid metabolism are involved in this effect. Thus, lenabasum induces the release of free arachidonic acid after activation of the CB_2_ receptor and phospholipase A_2_. Subsequently, increased concentrations of 15-deoxy-Δ^12,14^-PGJ_2_ are formed via COX-2, which leads to apoptosis and the subsiding of chronic inflammation via caspase-3 release. A LOX-mediated pathway is also thought to mediate the production of anti-inflammatory and proresolving lipoxin A_4_ and other proresolving mediators. However, the corresponding conditions of both possible signalling pathways and their interaction have not yet been clearly defined. PPARγ activation as a possible mechanism of anti-inflammatory action of lenabasum has been referred to earlier in this review [54].

Regarding the effect of cannabinoids on cancerous or precancerous lesions of the skin, some preclinical data have already suggested the potential of cannabinoids as preventive agents for skin cancer. For example, in a two-stage model of skin carcinogenesis in mice, in which skin carcinogenesis was induced by DMBA and promoted by TPA, the synthetic cannabinoid naphthoylindoles JWH-018, JWH-122, and JWH-210 were shown to reduce the promotion of TPA-induced carcinogenesis in mouse skin when applied topically 30 min before each TPA treatment. Thus, a topical chemopreventive effect for cannabinoids was demonstrated, at least in animal experiments [124]. However, this study did not investigate the role of cannabinoid receptors in the chemopreventive effects of cannabinoids. Regarding this aspect, UV irradiation has already been found to activate cannabinoid receptors, with UVB irradiation causing CB_1_ and CB_2_ phosphorylation and internalisation and subsequent activation of MAPK signalling pathways [125]. In the aforementioned study, CB_1_ and CB_2_ receptor double knockout mice showed less pronounced inflammation with lower activation of the transcription factor nuclear factor-κB (NF-κB), lower production of TNF-α and consequently a lower incidence of subcorneal pustules and epidermal erosions. Consistent with this finding, wild-type mice were four times more likely to develop papillomas than CB_1_ and CB_2_ double knockout mice. Inhibition of cannabinoid receptors could therefore be a prophylactic option to prevent skin cancer caused by UV radiation.

In the case of CBD, one study further showed that this cannabinoid increases the activity of antioxidant enzymes such as superoxide dismutase and thioredoxin reductase in keratinocytes exposed to UV radiation, suggesting that CBD may serve as a protective agent for skin cancer prevention [126]. The antioxidant and anti-inflammatory effects of CBD were later confirmed for skin fibroblasts in a 2D culture, but were less pronounced in a 3D culture [127]. A preventive effect of CBD on skin cancer demonstrated in nude rats irradiated with UVA and UVB light is that topical application of this substance to the skin resulted in a significant increase in phosphatidylethanolamines and ceramides, as well as the anti-inflammatory and proresolving mediator lipoxin A_4_, while proinflammatory and protumorigenic lysophospholipids, as well as PGE_2_ and thromboxane B_2_, were downregulated [128]. In summary, cannabinoids represent a chemically and functionally heterogeneous group of compounds that mediate potentially protective effects for the prevention of skin cancer through different mechanisms.

## 8. Clinical Trials on Cannabinoid Use in Cancer Patients 

The data currently available on the systemic effects of cannabinoids on carcinogenesis in cancer patients, and thus their benefits, are very limited. According to a 2006 published pilot study, intracranially administered THC was found to be safe in glioblastoma patients [129]. Furthermore, and comparatively more spectacularly, a recently published randomised, placebo-controlled phase Ib study in which nabiximols (oromucosal spray application of THC and CBD in a ratio of approximately 1:1) was administered to glioblastoma patients as add-on to temozolomide therapy showed a one-year survival rate of 83% in the nabiximols group versus a survival rate of 44% in the placebo group [130]. Notably, the latter study included only 21 patients. Interestingly, there are several other studies currently listed in the U.S. National Library of Medicine’s registry on the clinicaltrials.gov website that addressed the systemic effects of cannabinoids in cancer patients (for a review, see [131]). However, none of the registered studies focused on cannabinoids as a potential option for the systemic treatment of skin cancer. 

In a systematic review and meta-analysis of note, the quality of clinical studies on the use of cannabinoids for various medical indications was assessed with regard to chronic pain (28 studies, with 3 for cancer pain) and improvement of nausea and vomiting due to chemotherapy (28 studies) [132]. For the latter indication, cannabinoids were found to have a greater benefit than active comparators and placebo, but without reaching statistical significance in all studies. On the other hand, the studies were considered to provide only low-quality evidence. With regard to chronic neuropathic or cancer pain, the analyses revealed moderate-quality evidence that cannabinoids may be beneficial. In the latter analysis of the benefits and adverse effects of cannabinoids, it was shown that there is an increased cannabinoid-related risk of short-term but sometimes severe side effects, which include dizziness, dry mouth, nausea, fatigue, drowsiness, euphoria, vomiting, disorientation, drowsiness, confusion, loss of balance and hallucinations. The study also pointed out that there were no randomised clinical trials on the long-term side effects of cannabinoid therapies, which may include tumours, psychosis, depression or suicide, even when the literature search was extended to lower levels of evidence. Although the analysis of effects and side effects here is stratified according to the different cannabinoids and cannabinoid formulations, no reliable conclusions can yet be drawn about which cannabinoids are particularly suitable for clinical use in skin cancer.

Another recent systematic review of randomised controlled trials showed with moderate- to high-certainty evidence that compared with placebo, treatment with non-inhaled medicinal cannabis or cannabinoids results in a very small increase in the proportion of patients with chronic cancer and non-cancer pain who experience significant improvement in pain relief, physical functioning and sleep quality, along with some adverse side effects [133]. Finally, a recent review addressing the use of cannabis in cancer-related cachexia, including data from randomised controlled trials and non-randomised intervention trials, revealed no significant benefits of cannabinoids in terms of weight gain, appetite stimulation or improvement in quality of life [134].

It is also worth noting that in a study of cannabis use in patients with head and neck cancer undergoing radiotherapy, medical marijuana was reported to contribute to benefits in altered cognition, weight maintenance, depression, pain, loss of appetite, dysphagia, xerostomia, muscle cramps and sticky saliva [135]. However, the systemic effects on skin cancer were not investigated in this study. There was only one case report from 2006 dealing with the antiemetic effect of dronabinol in patients with distant metastatic malignant melanoma (stage IV). Here, treatment with dronabinol led to an improvement in the severity of symptoms from moderate and severe loss of appetite and nausea to only mild symptoms in five out of seven patients after four weeks of treatment [136].

In recent years, some clinical research has been conducted on the effects of cannabis use on chemotherapy outcomes. For example, a recent retrospective observational study showed a lower response rate of cancer patients with advanced melanoma, non-small cell lung cancer and clear cell renal carcinoma to treatment with nivolumab when cannabis was used concomitantly. Therefore, the use of cannabis in connection with immunotherapies has to be evaluated critically [137]. However, cannabis use in this study did not affect progression-free survival or overall survival. As limitations of the study design, the authors pointed out that it was a retrospective study that included only a small group of patients, dominated by a non-representatively high proportion of lung cancer patients, and that the follow-up period was very short. 

Another study reached a more concerning conclusion regarding the effect of cannabis use during immunotherapies. Here, cannabis reduced some of the adverse effects associated with immunotherapy, such as skin toxicity, colitis and thyroid disorders. On the other hand, cannabis users showed a less favourable prognosis in terms of time to tumour progression and overall survival than patients who did not use cannabis [138]. It is noteworthy that in this study, 54% of the patients had non-small cell lung cancer, and 37% of the patients had melanoma, all stage IV with metastases. The study again included a relatively small group of patients in terms of the different cancer types and the different oncological treatment lines, with large heterogeneity in the study populations, which was probably due to the fact that most patients switched cannabis products between months according to the cannabis companies’ recommendations. Finally, cannabis use was always less than 40 g per month, which is a small amount according to the Israeli Health Office.

Although not comparable with each other, there have also been studies that showed that marijuana use generally reduces the risk of cancer. For example, a population-based case-control study from 2009 found that 10 to 20 years of marijuana use was associated with a lower risk of head and neck squamous cell carcinoma after adjustment for potential confounding factors such as smoking and alcohol consumption [139]. Another population-based case-control study on the effects of marijuana use on the development of oral squamous cell carcinoma conducted several years earlier found that marijuana use was not associated with increased risk of this type of cancer [140]. Moreover, a pooled analysis of case-control studies provided data showing that regular marijuana use is not associated with squamous cell carcinoma of the head and neck or oral cavity cancer either, although low-strength evidence has suggested that marijuana smoking is associated with the development of germ cell tumours in the testes [141]. A recent meta-analysis examining the association between cannabis use and cancer incidence in the United States showed a trend towards a lower risk of cancer among cannabis users, with an overall risk of 0.90 (*p* = 0.065), which became statistically significant (0.86; *p* < 0.025) after removing the data on testicular cancer, which showed an increased risk [142]. Notably, this analysis excluded studies that did not adequately adjust for tobacco use.

In order to be able to make reasonable predictions about the systemic efficacy of cannabinoids, more prospective studies must be conducted in the future, which would then also allow statements about the correct application and dosage.

## 9. Outlook 

Currently, clinical data on the effect of cannabinoids on cancer progression from comprehensive clinical trials is lacking. Nevertheless, the data on the systemic anticancer effect of nabiximols in high-grade glioblastoma [130] also give some hope that this THC/CBD combination may be of benefit to patients as a possible option for other tumour entities, including skin cancer.

With regard to possible use of cannabinoid-based medicinal products in connection with skin cancer, different forms of application and indications of these substances have to be considered and discussed in a differentiated way. This concerns the systemic application of cannabinoids and the prophylactic topical application of cannabinoids for the prevention of sun-related and chemically induced skin cancers. Concerning the latter, the beneficial effects of CBD on reducing inflammation need to be further investigated in order to fathom the potential inhibitory effect of CBD on the development of skin cancer. On the adverse side effects of topical cannabinoid compounds in general, it should be noted that caution is also advised in eczema patients, as many topical cannabinoid-containing formulations contain a number of known irritants [143]. In fact, allergic sensitisation to cannabis is increasing and limiting the clinical use of this option [144].

In terms of the systemic effects of cannabinoids in the context of skin cancer, the preclinical data on the effect of CBD on melanoma [83] and squamous cell carcinoma cell proliferation [100], as well as on squamous cell carcinoma autophagy [101] and invasion [101], provide a relatively clear picture of a potential candidate for further investigation in clinical trials. However, the effect of CBD on autophagy and invasion of melanoma cells is not yet known. The effect of CBD on tumour angiogenesis in the context of melanoma and squamous cell carcinoma has not yet been addressed either, and reports of anti-angiogenic effects in skin cancer have been limited to a few studies reporting the anti-angiogenic properties of WIN 55,212-2 and JWH-133 [23,81] and the FAAH inhibitor URB597 [87]. A closer look at the literature also shows that a considerable number of publications have reported a potentially therapeutically useful effect of endocannabinoids on skin tumours. Thus, another way to control the growth and spread of skin cancer may lie in the enhancement of endocannabinoids at microenvironmental sites of tumour tissue by inhibiting endocannabinoid-degrading enzymes. In this context, the additional proapoptotic effect of AEA via the production of COX-2-dependent J-series PGs and PG ethanolamides shown in keratinocyte squamous cell carcinoma cells [95,96,97] may be a promising mechanism by which the efficacy of endocannabinoids and inhibitors of endocannabinoid turnover is enhanced in COX-2-overexpressing skin tumours. The fact that COX-2 activity also contributes to the anticarcinogenic effect of endocannabinoids in melanoma cells, as confirmed by other authors [86], is a further valuable piece of information. These preclinical findings could prospectively help to analyse patients’ tumours pre-therapeutically in order to selectively find patients with COX-2-overexpressing tumours as inclusion criteria for successful tumour treatment with FAAH inhibitors. To our knowledge, no study has yet investigated how the degradation products of 2-AG from the series of PG glycerol esters affect tumour growth. Under certain circumstances, such analyses could reveal an involvement of these eicosanoid metabolic pathways in the anticancer mechanisms of MAGL inhibitors.

As a matter of fact, cannabinoids could be promising drugs for the treatment and prophylaxis of skin cancer. However, in view of the recent observations mentioned above that cannabis use does not improve the efficacy of tumour immunotherapies but rather impairs it [137,138], it seems likely that the obvious combination of cannabinoids and checkpoint inhibitors is a rather unfavourable variant of combination therapy. This view is supported by the fact that the survival probability of patients using cannabis in combination with checkpoint inhibitors is approximately halved after 10 months of treatment [138]. In the context of currently applied therapies, however, it would be more conceivable for cannabinoids to be used in tumour therapies that do not involve checkpoint inhibitors.

A major threat to the potentially valuable therapeutic use of cannabinoids is that cannabis product providers are increasingly making and promoting numerous unsubstantiated claims to consumers [145], thereby distorting the true medical benefits of cannabinoids, including for the systemic and topical treatment and prophylaxis of skin tumours. Accordingly, false news about cannabis as a cure for cancer elicits tremendous interest, with high numbers of retweets on Twitter and engagements on Facebook compared with reactions to the news from leading cancer organisations [146]. As we noted some time ago, scientists need to gather clinical facts about the antitumour effects of cannabinoids before non-scientific sources create their own facts [22]. In this context, a recent study specifically pointed out the dangers of misinformation and misinterpreted claims about the efficacy of CBD for purposes such as curing or life-prolonging treatment of cancer [147]. According to the authors, misinformation published on fundraising platforms and additionally spreading via social networks requires transparent education, especially when it comes to life-saving treatment decisions. Unfounded exaggerated reporting aimed at maximising profits can lead people to avoid effective treatment because of mistrust of conventional therapies. This undermines public health systems and serious research and stands in the way of meaningful use of cannabinoid-based medicines in the long term, especially when unfounded overvaluation turns into ill will when patients’ expectations are not met.

## 10. Conclusions 

Preclinical results of recent years, especially with regard to antiproliferative and growth inhibitory effects, show that cannabinoids could be a promising option for the systemic treatment of melanoma and squamous cell carcinoma of the skin. Important questions in the clinical context will be which cannabinoid or combination of cannabinoids is actually most effective for which type of skin cancer and how high the dosage can be chosen without compromising patients’ quality of life through adverse drug effects. Another crucial question is in which combination with other cancer therapies cannabinoids show a synergistic anticancer effect and thereby possibly also reduce cytostatic-typical side effects and prevent chemoresistance. A consistent continuation of the previous preclinical studies as well as the implementation of randomised clinical studies on the effect of cannabinoids in skin cancer is encouraged on the basis of the data available so far.
